# Editorial: Epidemiology of antimicrobial resistance and virulence factors of emerging and re-emerging bacteria

**DOI:** 10.3389/fcimb.2024.1387087

**Published:** 2024-03-06

**Authors:** Gerardo Cortés-Cortés, Margarita M. P. Arenas-Hernández, Manuel G. Ballesteros-Monrreal, Rosa del Carmen Rocha-Gracia, Edwin Barrios-Villa

**Affiliations:** ^1^ Posgrado en Microbiología, Centro de Investigaciones en Ciencias Microbiológicas, Instituto de Ciencias, Benemérita Universidad Autónoma de Puebla, Puebla, Mexico; ^2^ Department of Microbiology and Environmental Toxicology, University of California at Santa Cruz, Santa Cruz, CA, United States; ^3^ Departamento de Ciencias Químico-Biológicas y Agropecuarias, Universidad de Sonora, H. Caborca, Sonora, Mexico

**Keywords:** antimicrobial resistance (AMR), virulence factors, mobilizable genetic elements, emerging pathogens, virulence-resistance co-occurrence

The World Health Organization has declared antimicrobial resistance (AMR) a concerning top global public health issue that is estimated to result in significant additional healthcare costs, hospitalizations, and deaths, affecting millions of people by 2050 (Antimicrobial Resistance Collaborators, 2022). AMR is a phenomenon that has been evolving through time due to the participation of mobilizable genetic elements (MGEs), including plasmids, transposons, and integrons, which accelerate the horizontal spread of antibiotic resistance genes (Sultan et al., 2018). This Research Topic compiled information about the prevalence of virulence and resistance determinants in clinically important bacteria such as *Escherichia coli*, *Klebsiella pneumoniae*, *Staphylococcus aureus, Salmonella*, *Acinetobacter baumannii*, and *Streptococcus pneumoniae*, as well as emerging pathogens such as *Staphylococcus epidermidis*, *Staphylococcus warneri, Mycobacterium fortuitum*, and *Parvimonas micra.*

AMR represents a public health concern affecting whole living beings that needs to be analyzed from the one health approach, in which comprehensive human activities influence animal and environmental health and *vice versa*. Based on this perspective, Gonzales-Zubiate et al. observed the occurrence of *bla*_CTX-M_ resistance genes from the variants CTX-M-55 and -65 in *E. coli*, and CTX-M-14 and -27 in *K. pneumoniae* in companion animals from Ecuador; this study highlights the identification of sequence types that might be clonally expanded. The tracking of bacterial clones has been improved by implementing novel tools such as whole-genome sequencing (WGS), which also highly contribute to the study of MGEs. Using this technology, Neidhöfer et al. obtained important data on the distribution of the AMR genes and sequence types of bacteria implicated in bloodstream infections; they predominantly identified *Enterococcus faecalis, Enterococcus faecium, S. aureus, S. pneumoniae, Streptococcus pyogenes, Bacteroides fragilis, E. coli, K. pneumoniae, Enterobacter* spp, *Citrobacter* spp, *Proteus mirabilis, Serratia marsescens, A. baumanii, Pseudomonas aeruginosa*, and *Stenotrophomonas maltophilia*. Additionally, Jiménez-Rojas et al. studied Extended-Spectrum Beta-Lactamase (ESBL)-*E. coli* and ESBL-*K. pneumoniae* colonization in a Mexican neonatal intensive care unit, revealing a high prevalence (87%) and a relationship with healthcare-associated infections (22%). Variable resistance to cephalosporins, susceptibility to carbapenems, and genetic clonality underline the importance of addressing the search and study of ESBL-producing Enterobacterales in neonatal care. In this sense, another bacterium that has become one of the major causes of nosocomial infections is *A. baumannii*. Fernández-Vázquez et al. investigated the multidrug resistance profile of sequenced strains of *A. baumannii* in Mexican hospitals, identifying diverse resistance genes, including the clinically significant OXA-72 carbapenemase-encoding gene, highlighting the high adaptation of *A. baumannii* to new and variable niches. This study emphasized the role of antimicrobial resistance plasmids in disseminating resistance genes, which is crucial for effective control strategies. In the community setting, *Salmonella* is a concerning bacterium linked to outbreaks. Ma et al. analyzed 19 non-typhoidal *Salmonella* isolates, which were subjected to serovar identification, antimicrobial susceptibility testing, and biofilm formation evaluation. WGS revealed genetic relatedness, especially among *Salmonella* serovar Mbandaka isolates, suggesting the existence of a persistent source in China for more than 5 years, mediated by resistance mechanisms such as plasmids harboring *bla*_CTX-M-15_ and virulent *mrkABCDF* operons. On the other hand, Balbuena-Alonso et al. evidenced new routes of pathogenic and multidrug-resistant bacteria transmission through food products, highlighting the role of agricultural products as reservoirs for resistance and virulence genes. This group characterized the A23EC *E. coli* strain from spinach, which belongs to the C2b sublineage of ST131, harboring three pathogenicity islands, a chromosomally integrated transposon (TnMB1860) associated with six resistance genes, and a conjugative IncF plasmid carrying a tetracycline resistance gene. Even when vaccines are available to protect against bacterial infections, AMR is a concerning issue. In a systematic review conducted by Sandoval et al., AMR in *S. pneumoniae* from invasive disease in Latin America and the Caribbean was assessed. Of 8,600 records, 103 studies were included, covering 49,660 positive samples. Penicillin resistance was observed in 21.7% of the isolates, with higher rates in children aged 0 to 5 years. Ongoing surveillance is crucial for monitoring evolving serotypes and AMR patterns, especially after the introduction of conjugated pneumococcal vaccine.

The role of emergent bacteria causing uncommon and hard-to-diagnose infections contributes to the limitation of effective treatment options. The case report by Shao et al. informs the diagnosis and treatment of *P. micra* (a Gram-positive anaerobic bacterium that exhibits colonization tendencies on oral mucosal and skin surfaces) in a 53-year-old male with recurrent hemoptysis. *P. micra* was identified using metagenomic next-generation sequencing, reflecting the importance of incorporating last-generation technologies as diagnostic tools. This infection was treated successfully with a combined beta-lactam and fluoroquinolone therapy, evidencing the importance of efficient treatment alternatives even for atypical infections.

Identifying genes associated with AMR in emerging pathogens remains a major issue. Alam et al. reported the isolation and genomic characterization of a highly MDR strain of *M. fortuitum* from a pulmonary infection harboring several genetic determinants of resistance, including acquired ones and associated mutations; interestingly, the strain investigated identified virulence genes also found in *M. tuberculosis* and *Mycobacterium abscessus*.

MGEs also harbor virulence genes that might have health implications for the host, contributing to biological variability but also representing a therapeutic challenge due to the emergence of pathogenic multidrug-resistant strains. Amer et al. investigated coagulase-negative staphylococci (CoNS) in acne lesions in Egypt, identifying *S. epidermidis* as the predominant species. Isolates from severe acne cases exhibited high antibiotic resistance and strong biofilm formation. WGS revealed multidrug resistance and virulence genes, highlighting the potential pathogenicity of CoNS in acne infections, especially the emergence of *S. warneri*, a previously reported uncommon pathogen.

In conclusion, this Research Topic contributes valuable information about the spread of AMR in emergent and re-emergent pathogenic bacteria, which is accelerated by the horizontal transfer of MGEs, reducing the efficiency of antibiotics used to control infections in humans and animals in clinical and environmental settings. Genomic-based screening strategies and new-generation technologies are prominent tools for gaining insight into the proper treatment, prevention, and control of hospital- and community-acquired diseases caused by multidrug-resistant bacteria.

## Author contributions

GC-C: Investigation, Writing – original draft, Writing – review & editing. MMPA-H: Investigation, Writing – original draft, Writing – review & editing. MGB-M: Investigation, Writing – original draft, Writing – review & editing. RCR-G: Investigation, Writing – original draft, Writing – review & editing. EB-V: Investigation, Writing – original draft, Writing – review & editing.
